# Acknowledgment to the Reviewers of *Advances in Respiratory Medicine* in 2022

**DOI:** 10.3390/arm91010002

**Published:** 2023-01-18

**Authors:** Advance in Respiratory Medicine Editorial Office

**Affiliations:** MDPI AG, St. Alban-Anlage 66, 4052 Basel, Switzerland

High-quality academic publishing is built on rigorous peer review. *Advances in Respiratory Medicine* was able to uphold its high standards for published papers due to the outstanding efforts of our reviewers. Thanks to the efforts of our reviewers in 2022, the median time to first decision was 23 days and the median time to publication was 64 days. Regardless of whether the articles they examined were ultimately published, the editors would like to express their appreciation and thank the following reviewers for the time and dedication that they have shown *Advances in Respiratory Medicine*:
Aggeliki VerveniotiKrzysztof FilipiakAlok KumarLee NguyenAmanda Iglesias‪Łukasz MokrosAnand ShahMaciej Iek SkrzypczakAndras BikovMałgorzata PacAngel-Augusto Perez-CalatayudMałgorzata SobieckaAnna BręborowiczMałgorzata ZnykArvind Kumar ShuklaMalik SallamAthanasia ProklouMarcin KurzynaBogdan JakielaMarek NiedoszytkoChih-Cheng HuangMaribel Isabel Botana-RialChristelle CorauxMario Josue Valladares-GarridoClaire L. JacksonMariusz ChabowskiClaudio TirelliMarousa KouvelaDaniel HackettMaulin PatelDaniel PintoMilosz JankowskiDavid Chávez-FloresMohammad ShadabDawid WnukMonika FranczukDenisse ParraMusaddique HussainDominika ZającNicolae GicaDushyant DamaniaPatrizia ZaramellaEdyta Heropolitanska-PliszkaPawel KucaElisabeth M. BendstrupPietro AlfanoEnrique CarreroPrachi SalujaErica BazzanPrasanth BalasubramanianFaranak RafieeRavindra A. PrabhuFilippo Tommaso GallinaRiccardo PozzanGino Seravalle SeravalleRitesh AgarwalGrzegorz DziubanekRobert HopeflHenryk MazurekSanthosh RajamaniHongran YinSayed AzeezJacek NasiłowskiStefan WesołowskiJedrzej ChrzanowskiSvetlana V. GuryanovaJerzy MarczakSyed N. U. A. KirmaniJosé Norberto Sancho-ChustSzczepan CoftaKaj BloklandTadeusz PrzybyłowskiKata CsekoTatsuhiko FurukawaKatarzyna Błasińska-PrzerwaTerenzio CosioKeigo UchimuraValeria ViscoKenneth Wayne AltmanVicente Javier Clemente-SuárezKerry L. TuckerVivian TullioKillen Harold Briones ClaudettWojciech PiotrowskiKoji KomoriZhen ShuKonlawij TrongtrakulZia Hashim


